# Unqualified Practice

**Published:** 1908-12-05

**Authors:** 


### The Hospital

A Journal of

The Medical Sciences and Hospital Administration.

New Series. No. 91, Vol. IV. [No. 1163, Vol. XLV.] SATURDAY, DECEMBER 5, 1908.

### UNQUALIFIED PRACTICE.

Among the many fundamental misconceptions of the art of healing as practised by the medical profession is that idea, so widespread among the public that it seems almost hopeless to attempt its uprooting, of something secret and mysterious about the treatment of diseases. This notion may be supposed to be the heritage of the medieval magic-mongering which usurped the place of more legitimate therapeutics: at all events it seems difficult to suggest any other hypothesis by which to account for it. It is sufficient for any man, however ignorant and uneducated, to announce loudly enough and often enough that he has some secret panacea, some mysterious gift of manipulation, or what not; and he will, if he possess a certain kind of shrewd cunning, not merely make a fortune, but obtain also a devoted following of enthusiastic proselytes. Curiously enough the laity pay little or no attention to diagnosis. Anyone—the patient himself or an officious friend—is considered capable of that; whereas treatment is regarded as the gift of individuals, and even as innate in them. It might be thought that anyone who has ever passed under the care of a capable and painstaking medical man can appreciate the minute care which is exercised to eliminate error in diagnosis, even though the mental processes of the doctor and the meaning of the methods employed are incomprehensible. But in point of fact it is not so, and any medical man, even any quack, is supposed to be capable of diagnosing disease on sight of the patient. It is these two conceptions, both erroneous to the most ridiculous degree, which account for the attitude of the public towards unqualified practice and the endeavours of the medical profession to mitigate, in the interest of the State and the community, some of the grosser evils associated with it.

What some of these evils are was explained at length last week to the members of the Medico-Legal Society in meeting assembled by Mr. A. G. Bateman, the well-known Secretary of the Medical Defence Union, who is especially qualified to speak upon this subject. We will venture to say that even medical men must have been astounded as they listened to the catalogue of unpunished, and in the present state of the law unpunishable, frauds and impostures. It is not merely that unqualified practice is freely permitted in this country; that may or may not be in the interests of the State: but Parliament has definitely decided that any unqualified person may undertake the treatment of disease provided he makes no claim to be a duly registered practitioner. We, as a profession, most of us regard this state of things as calamitous, not from our own point of view, but from that of the national welfare, and we have a right to express our opinion to that effect. But over and above this it is possible by a very moderate degree of astuteness for any quack to delude the public into supposing that he is properly qualified and registered, and yet to evade the penalties intended by the law for this offence. This is not only possible, it is being done every day. A judge of the High Court has held that the law on the subject "is in a complete fog"; yet another has actually expressed from the Bench his opinion that some quacks know more than doctors. Between one thing and another, judicial ignorance and legal subtlety, can it be wondered at that the meshes of the law permit the escape of quite big fishes as well as the smaller fry of fraudulent medical humbugs?

The present state of the law, as interpreted by the judges, allows ex-convicts to conduct unqualified practice without let or hindrance, to assume such titles as M.D. without effective interference, and, as Mr. Bateman showed, to utilise the confidence of the public in the profession to which they thus pretend to belong so as to further all sorts of crimes and swindles quite unconnected with medicine and surgery. It stultifies the provisions of the various Acts passed in recent years to protect and secure the public health by permitting registrars, who have no medical knowledge whatever, to accept death certificates from anyone who is educated enough to write his own name; a permission which is so freely taken advantage of in some parts of England that 5 per cent. of the certificates there accepted are totally valueless. It countenances the extraordinary anomaly that dogs and horses are better, far better, protected from incompetent advice during sickness than are human beings. And, lastly, the law cannot even remain consistent in inconsistency, for it has lately been ordained that no woman may be attended in childbirth after 1910 except by a medical man or qualified midwife. We hear a good deal about man-made laws as applied to women; it seems in one respect at any rate that they afford women justice and mercy which are denied to men.

What Mr. Bateman demands is, to put it briefly, that the law shall put doctors into a position as secure as that of the lawyers. While we applaud the suggestion, it is probably not a practicable one, or not as yet. One of the lawyers who took part in the discussion on the paper explained how, in his view, the parallel which Mr. Bateman had drawn fell short of completeness. He held that medicine, being still a progressive science in which important discoveries are still being and to be made, it is essential that no restraint should be put upon such discoveries by restricting in any way the practice of that science. If this view is widely held, as is quite likely, it merely shows what radical misconceptions still exist about medicine. It presupposes that scientific discoveries are hit upon at random, much as a man may find a shilling in the street, whereas in reality the process is a long and arduous one, involving as a rule years of work of highly developed intellects in those who have been especially trained for such research. The total prohibition of unqualified practice is an integral part of any complete scheme of hygienic reform; but for the present we shall have to be content with remedying some of its more glaring evils.

